# Multidisciplinary approach to connective tissue disease (CTD) related pleural effusions: a four-year retrospective evaluation

**DOI:** 10.1186/s12890-019-0919-2

**Published:** 2019-08-27

**Authors:** Hugh Ip, Parthipan Sivakumar, Eugene Ace McDermott, Sangita Agarwal, Boris Lams, Alex West, Liju Ahmed

**Affiliations:** Guy’s and St Thomas’ NHS Foundation Trust, Westminster Bridge Road, London, SE1 7EH UK

**Keywords:** Pleural effusion, Pleura, Thoracoscopy, Connective tissue disease

## Abstract

**Background:**

CTD-related pleural effusions are rare and challenging to diagnose. Our lung inflammation service (with expertise in rheumatology, interstitial lung disease and respiratory failure) works closely with the pleural team. This study aims to review the multidisciplinary approach to CTD-related pleural effusions at a tertiary centre.

**Methods:**

All patients with CTD-related pleural effusions at St Thomas’ Hospital, London were included. Retrospective data were collected from Dec 2013 to 2016.

**Results:**

The lung inflammation service performed an expert clinical assessment and targeted investigations. 11 patients (ages 23–77) were identified with CTD related pleural disease. 9 (82%) patients were given a new CTD diagnosis, with pleural disease as the first manifestation. The range of conditions were: rheumatoid arthritis [3] ,IgG4-related disease [2] ,adult Still’s disease [2] ,vasculitis [1] ,SLE [1] ,drug-induced lupus [1] ,and Behcet’s [1].

The pleural team review took place 1 day (median) after referral. 73% of diagnoses (8 patients) were achieved with local anaesthetic pleural interventions (a combination of: aspiration, drain, or percutaneous biopsy). This included 1 patient who required no pleural intervention. 1 required medical thoracoscopy, and 2 underwent thoracic surgery.

Diagnoses were made by integrating all available evidence such as clinical assessment, imaging, and autoimmune serology. No diagnosis was achieved by pleural cytology or histology analysis alone.

8 (73%) were commenced on prednisolone acutely (vasculitis, SLE, drug-related lupus, 1 patient with rheumatoid arthritis, Behcet’s, 2 patients with Adult Still’s disease, 1 patient with IgG4-related disease). Of these 8, one patient with rheumatoid arthritis received IV methylprednisolone beforehand, one patient with IgG4-related disease was weaned off prednisolone to methothrexate, two patients with Adult Still’s disease were on colchicine as well, and one patient with Behcet’s was on cyclophosphamide as well. 7 (64%) were managed as outpatients; 4 required admission. The median time from pleural review to diagnosis was 53 days.

**Conclusions:**

Diagnosis can be challenging in patients presenting with pleural disease as the first manifestation of a CTD. We recommend a multidisciplinary approach in management.

## Background

A new pleural effusion may be caused by a wide range of conditions. The British Thoracic Society produced guidelines in 2010 recommending a systematic approach to achieve a diagnosis, aiming to streamline investigations and interventions [1]. Since then, the evidence base for managing malignant and infective effusions has developed through a series of clinical trials. In comparison, research on benign non-infective pleural effusions has been more limited [2]. A number of these are caused by connective tissue diseases (CTD).

Pleural effusions from CTD are caused by increased capillary permeability, as extravascular fluid moves from the lung’s interstitium, across the mesothelium into the pleural space [3]. This may be due to a number of reasons [4], such as a pleural infiltrative process. In addition, circulating immune complexes that localise to the pleura, can activate the complement system causing endothelial injury. Enzyme and free radical release from white blood cells also accentuate the inflammatory process.

CTD-related pleural effusions are rare and challenging to diagnose. The most common CTDs to affect the pleura are rheumatoid arthritis and systemic lupus erythematosus (SLE) [1]. A prospective observational cohort study over 7 years at a specialist pleural unit identified 356 nonmalignant pleural effusions. 9.8% of these were given a diagnosis of inflammatory pleuritis, and 7.6% attributed to other diagnoses (including chylothorax, rheumatic causes, trauma, and drug-induced causes) [5].

The same unit described the value of a pleural specialist team to improve the efficacy and efficiency of managing patients with pleural disease [6]. In most pleural teams, the medical specialties involved are commonly: respiratory, oncology and palliative care. However, the pleural team in our centre works closely with the lung inflammation service, with expertise in rheumatology, interstitial lung disease and respiratory failure. This study aims to review the multidisciplinary approach to CTD-related pleural effusions at a tertiary centre. To our knowledge, there is no published evidence describing a multidisciplinary approach to CTD-related pleural effusions.

## Methods

### Study design

This study is a retrospective evaluation of cases. We reviewed electronic hospital records, imaging, blood tests, pleural fluid analysis and pleural biopsy analysis. Data were collected relating to CTD diagnosis, pleural and surgical interventions, and CTD specific systemic therapy.

As a retrospective service evaluation, written patient informed consent and regional ethics approval was not required.

### Participants

All patients diagnosed with CTD-related pleural effusions at St Thomas’ Hospital, London were included. We included patients referred to pleural clinic between November 2012 and 2016. These patients are usually referred by the general medical or rheumatology teams for a specialist pleural opinion when the etiology of the pleural effusion is unclear, or where there is an acute clinical concern. We excluded patients with a CTD attending pleural clinic with a pleural effusion due to other (non-CTD related) etiologies.

### Interventions

Pleural aspirations, drains, biopsies and medical thoracoscopies were performed in the pleural clinic at St Thomas’ Hospital in accordance with national guidelines [7, 8]. Out of hours, aspirations and drains were performed by the radiology department.

### Assessments

Statistical analysis was carried out using Microsoft Excel. Descriptive statistics were used to evaluable the service, and to summarise the clinical characteristics of the subjects.

## Results

Eleven patients (ages 23–77) were identified with CTD related pleural disease (Table 1). They were seen by the lung inflammation service, who performed an expert clinical assessment and targeted investigations, usually after review by the pleural team. 9 (82%) patients were given a new CTD diagnosis, with pleural disease as the first manifestation. The range of conditions were: rheumatoid arthritis [3], IgG4-related disease [2], adult Still’s disease [2], vasculitis [1], SLE [1], drug (carbamazepine)-induced lupus [1], and Behcet’s [1].

**Table 1:** Patient demographics

Characteristic	*n* (%)
Gender (*n* = 11)	
Male	8 (73)
Female	3 (27)
Age range	23–77
Median age	50
CTD diagnosis	
Rheumatoid arthritis	3 (27)
IgG4-related disease	2 (18)
Adult Still’s disease	2 (18)
Vasculitis	1 (9)
SLE	1 (9)
Drug-induced lupus	1 (9)
Behcet’s	1 (9)

The pleural team review took place 1 day (median) after referral. 73% of diagnoses (8 patients) were achieved with local anaesthetic pleural interventions (a combination of: aspiration, drain, or percutaneous biopsy). This included 1 patient who required no pleural intervention. 1 required medical thoracoscopy, and 2 underwent thoracic surgery (Table 2). The patient who underwent a medical thoracoscopy (for pleural thickening) after an aspiration, had a final diagnosis of a rheumatoid arthritis associated pleural effusion. One patient who underwent thoracic surgery was referred directly to the surgical team, subsequently requiring a pericardiectomy for a diagnosis of IgG4-related disease. Another patient underwent VATS to rule out a pleural malignancy, before commencing treatment for a pleural effusion related to drug-induced lupus.

**Table 2:** Pleural/surgical interventions

Pleural intervention	*n* (%)
None	1 (9)
Pleural aspiration only	3 (27)
Chest drain only	2 (18)
Pleural aspiration and chest drain	1 (9)
Pleural aspiration and biopsy	1 (9)
Pleural aspiration and biopsy, drain and VATS	1 (9)
Pleural aspiration and medical thoracoscopy	1 (9)
VATS, thoracotomy, pericardiectomy	1 (9)

Table 3 illustrates how diagnoses were made by integrating and analysing all available evidence, to include clinical assessment, imaging, and autoimmune serology. No diagnosis was achieved by pleural cytology or histology analysis alone. The pleural fluid pH was not measured for these patients (although it is available at our centre), as pleural infection was low on the list of differentials.

**Table 3:** Key clinical information of patients

Connective tissue disease diagnosis	Presenting symptoms / Autoimmune serology	Evidence for diagnosis*	Pleural fluid glucose (mmol/L)	Pleural fluid LDH (IU/L)	Pleural fluid protein (g/L)	Pleural micro	Pleural fluid appearance	Pleural fluid cytology	Histology	CT chest findings	Outcome
Rheumatoid arthritis	Pleuritic chest pain, dyspnoea, polyarthralgia, fatigue anti-CCP + ve	Clinical assessment, autoimmune serology	3.4	794	55	Fluid MCS/AFB neg Biopsy AFB neg	Turbid yellow fluid	2/3/16 Neutrophils, lymphocytes, macrophages, prominent eosinophils. Few reactive mesothelial cells. 23/3/16 Mixed lymphoid cell population; plasma cells and eosinophils. Occasional mesothelial cells.	Pleural biopsy: moderate acute and mild chronic inflammation, surface mesothelial denudation, macrophages and fibrin.	Bilateral hilar lymphadenopathy. Left-sided pleural effusion and bibasal groundglass change with intralobular septal thickening demonstrated.	Resolution of pleural effusions on CXR by week 7 on weaning prednisolone (after pulsed methylprednisolone)
Rheumatoid arthritis	Fever, cough, fatigue, pleuritic chest pain, dyspnoea anti-CCP and RhF + ve	Previous CTD diagnosis, clinical assessment, autoimmune serology	None	None	None	n/a	n/a	None	None	Pericardial hyperattenuation, either in keeping with thickening and low-grade enhancement or a complex pericardial effusion. Shallow left pleural effusion.	Resolution of pleural effusions 5 months after pleural review (one prior week of prednisolone)
Rheumatoid arthritis	Cough, fever anti-CCP and RhF + ve	Previous CTD diagnosis, clinical assessment, pleural fluid glucose, pleural biopsy	0	5947	55	Fluid MCS/AFB neg Biopsy AFB neg	Turbid yellow fluid	Lymphocytes and macrophages and scattered histiocytic multi-nucleate giant cells	Pleural biopsy: fibrous tissue with a dense lymphoplasmacytic infiltrate. Suggestion of histiocyte palisading; possibility of rheumatoid nodule, but insufficient for definitive diagnosis.	Moderate right pleural effusion. A calcified granuloma is noted in the right lower lobe. Minor right subpleural nodularity is seen. Emphysema.	Pleural effusion stable 1 year after discharge from pleural clinic (during which 5 mg prednisolone daily started for joint pains)
IgG4-related disease	Dyspnoea, cough, oedema IgG4 2.71 (0.23–1.11 g/L) IgG1 11.3 (4.8–9.5 g/L)	Pericardiectomy histology, autoimmune serology	None	None	None	Fluid MCS/AFB neg Biopsy AFB/MCS neg	Bloodstained fluid	A few benign mesothelial cells (Pleural biopsy: mildly inflamed pleural tissue. Single non-necrotising granuloma.)	Pericardial biopsy: dense keloid-like fibrosis. Foci of chronic inflammation and in these IgG4 plasma cells comprise a high population of IgG+ plasma cells.	Right pleural effusion and ascites. Cervical and mediastinal lymphadenopathy. Pericardial calcification and thickening suggest previous pericardial effusion/pericarditis.	Pleural effusion improved over 2 months (prednisolone started then weaned off onto methotrexate)
IgG4-related disease	Dyspnoea, dizziness IgG4 1.53 (0.23–1.11 g/L) IgG1 12.4 (4.8–9.5 g/L)	Pericardiectomy histology, clinical assessment, autoimmune serology	None	27	6	Fluid Scanty growth *Staphylococcus aureus*, AFB neg	Slightly cloudy yellow fluid	Mesothelial cells and mixed inflammatory cells (predominantly neutrophil polymorphs).	Pericardial biopsy: Diffuse moderate to severe fibrosis associated with focal calcification and mild chronic inflammatory cell infiltrate. IgA shows non-specific patchy mild staining.	Small bilateral pleural effusions. No pericardial effusion although some pericardial calcification is noted. Likely reactive mediastinal nodes.	Left pleural effusion resolved and small right pleural effusion 1 year after pleural review (started on furosemide)
Adult Still’s disease	Fever, cough, dyspnoea, chest pain, myalgia, sore throat, rash	Clinical assessment, raised ferritin	10.3	894	33	Fluid MCS/AFB neg	Straw coloured fluid	Acute inflmmatory cells. A few reactive mesothelial cells, histiocytes and lymphocytes.	None	Small bilateral pleural effusions with bibasal collapse/consolidation. Moderate pericardial effusion.	Pleural effusions resolved after 1 month (weaning prednisolone and colchicine)
Adult Still’s disease	Pleuritic chest pain, dyspnoea, fever, sore throat, arthralgia	Clinical assessment	None	None	34	Fluid MCS/AFB neg	Cloudy yellow fluid	Numerous neutrophils (95%) with occasional macrophages and mesothelial cells.	None	Small bilateral pleural effusions and a trace of pericardial fluid.	Pleural effusions resolved after 2 months (weaning prednisolone and colchicine)
Vasculitis	Dyspnoea, weight loss, appetite loss P-ANCA, Anti-MPO 222 (0-10 U/ml)	Clinical assessment, autoimmune serology, CT showing adrenal infarcts	6.9	279	48	Fluid MCS/AFB neg	Bloodstained fluid	Mixed lymphoid cell population along with neutrophil polymorphs, histiocytes and very occasional mesothelial cell.	none	Bilateral calcified pleural plaques. Right pleural thickening and effusion. Emphysema. Multiple solid subpleural and peri-fissural bilateral nodules	Pleural effusion resolved after 5 months (4 month course of weaning prednisolone)
SLE	Pleuritic chest pain, dyspnoea Anti ds-DNA 415 (0-10 IU/ml), anti-Smith +ve	Previous CTD diagnosis, clinical assessment	5.2	304	59	Fluid MCS/AFB neg	Cloudy light brown fluid	Mesothelial cells, macrophages, neutrophils and lymphocytes.	none	None	Pleural effusion resolved after 7 months (prednisolone weaned to maintenance)
Drug-induced lupus (cabamazepine)	Dyspnoea, rash, ulceration of hands and feet ANA +ve, ENA RNP + ve, ENA SSA (Ro) + ve, ENA Sm + ve ANCA neg	Clinical assessment, autoimmune serology	6.5	228	47	Fluid MCS/AFB neg Biopsy AFB/MCS neg	Turbid orange fluid	16/11/12 Reactive mesothelial cells, polymorphs, lymphocytes and histiocytes. 5/12/12 Numerous mesothelial cells, macrophages and a mixed population of lymphocytes. 11/12/12 Histiocytes, a few lymphocytes and reactive mesothelial cells. 18/12/12 Reactive mesothelial cells, lymphocytes and histiocytes. 27/12/12 Histiocytes, lymphocytes, reactive mesothelial cells, neutrophils, plasma cells. 13/1/13 Blood only.	16/11/12 pleural biopsy: Single focus of large cells at the edge of one fragment, most likely reactive mesothelial cells. Nuclei are pleomorphic, irregular nuclear membranes and nuclear chromatin clearing. 13/1/13 pleural biopsy: Pleura markedly oedematous; granulation tissue at the surface, scattered foci of chronic inflammatory cells.	Left pleural effusion with minimal nodularity and mild volume loss in the left hemithorax. Shallow pericardial effusion. Widespread mild lymphadenopathy.	Pleural effusion resolved after 9 months (prednisolone weaned to maintenance)
Behcet’s	Chest tightness	Clinical assessment	4.8	380	44	Fluid MCS/AFB neg	Turbid bloodstained fluid	Numerous lymphocytes, histiocytes and blood.	none	Large right main pulmonary artery thrombus. Multiple left sided pulmonary emboli. Bilateral pleural effusions with consolidaton/atelectasis. 2 small round pulmonary foci and a band of consolidation in the right upper lobe. Wedge-shaped consolidation in the middle and right lower lobes.	Pleural effusions resolved after 1 year (cyclophosphamide and weaning prednisolone)

*These were the key factors used by the mutldisciplinary team in securing a diagnosis

Eight (73%) were commenced on prednisolone acutely (vasculitis, SLE, drug-related lupus, 1 patient with rheumatoid arthritis, Behcet’s, 2 patients with Adult Still’s disease, 1 patient with IgG4-related disease). Of these 8, one patient with rheumatoid arthritis received IV methylprednisolone beforehand, one patient with IgG4-related disease was weaned off prednisolone to methothrexate, two patients with Adult Still’s disease were on colchicine as well, and one patient with Behcet’s was on cyclophosphamide as well. 7 (64%) were managed as outpatients; 4 required admission. The median time from pleural review to diagnosis was 53 days.

## Discussion

In the work up for interstitial lung disease (ILD), assessment by a rheumatologist is recommended in suspected CTD [9]. The input of a rheumatologist is invaluable in providing an expert clinical review, then directing and interpreting autoimmune testing. Our centre has found this to be the case – in managing CTD-related pleural effusions. The lung inflammation service was set up to assist the critical critical care team in managing severe respiratory failure; they now work closely with the pleural service as well. To our knowledge, this is the first published evaluation of a collborative approach for inflammatory pleural disease. Case reports of CTD-related serositis do not describe a similarly coordinated approach [10–12]. Our results suggest that the value of pleural fluid and tissue analysis is to exclude common conditions such as malignancy and infection, while a multidisciplinary approach integrating all available diagnostic information is needed for complex cases such as CTD related pleuritis.

In this service evaluation, the multidisciplinary approach to CTD-related pleural effusions has demonstrated efficiency in achieving a diagnosis in a median of 53 days from the first review by the pleural team. Pleural procedures were streamlined, with 73% of diagnoses being achieved by local anaesthetic interventions. 64% of cases were managed in the outpatient setting. Published data describe the outcomes of pleural service outcomes as a whole [13, 14]. This is the first study to focus on the management of CTD-related pleural effusions.

The study was limited by the small number of cases, due to the rareity of CTD-related pleural effusions. A multi-centre collaboration to establish a larger database would facilitate advancement in best managing this complex patient cohort.

## Conclusions

Diagnosis can be challenging in patients presenting with pleural disease as the first manifestation of a CTD. We recommend a multidisciplinary approach in management.

## Data Availability

Due to our local governance policy, we do not have permission to make the data sets on which the conclusions of the paper rely publicly available. A truncated data set (removing all potentially identifying features) may be made available on an individual request basis.

## Abbreviations

CTD: connective disease disease
ILD: interstitial lung disease
SLE: systemic lupus erythematosus
VATS: video-assisted thoracoscopic surgery
